# Supplementary material for: SARS-CoV-2 transmission and impacts of unvaccinated-only screening in populations of mixed vaccination status
Source: Nat Commun. 2022 May 19;13:2777. doi: 10.1038/s41467-022-30144-7 (PMC9120147; doi:10.1038/s41467-022-30144-7)
Supplement: Supplementary file 1 — Supplementary Information [file 41467_2022_30144_MOESM1_ESM.pdf]

SARS-CoV-2 Transmission and Impacts of Unvaccinated-Only Screening Testing in Populations of Mixed Vaccination Status: Supplementary Materials

Kate M. Bubar*, Casey E. Middleton*, Kristen K. Bjorkman, Roy Parker, Daniel B. Larremore

Contents of This File:

- Supplementary Table 1 - Summary of population, infection, and testing parameters used in modeling and simulation.
- Supplementary Table 2 - Summary of immunity parameters used in modeling and simulation.
- Supplementary Figures 1 - 8.
- Supplementary Text - Derivation of effective reproductive number R_{eff}

**Parameter**	**Description**	**Value**	**Reference**
Population parameters			
N	Population size	20,000	—
ϕ	Proportion of population vaccinated	$[0, 1]$<br>US: 0.58	[24]
ψ	Proportion of population with infection-acquired immunity	$[0, 1]$<br>US: 0.35	[54]
Infection parameters			
σ^{-1}	Latent period	3 days	[55]
γ^{-1}	Infectious period	6 days	[56]
R_0^{NPI}	Basic reproductive number	$\{4, 6\}$	see Methods
α	Probability of transmission given contact (tuned to achieve the desired R_0^{NPI})	$R_0^{\text{NPI}}\gamma/N$	—
IHR	Infection hospitalization rate for naive unvaccinated	delta: 0.02<br>omicron: 0.01	[57, 58]<br>[59, 60]
Testing parameters			
θ	Fraction by which screening & isolation reduces typical *unvaccinated* infectious period*		
	no screening	0	—
	weekly screening, 50% compliance	0.242	[7]
	weekly screening, 99% compliance	0.473	[7]
	2× weekly screening, 99% compliance	0.808	[7]

Supplementary Table 1: **Summary of population, infection and testing parameters used in modeling and simulation.**
*Assuming PCR testing with a one day turnaround time for test results.

Parameter	Description	Value	Reference
Immunity parameters			
VE_S	Vaccine effectiveness to decrease susceptibility to infection	**delta**<br>waning = 50%<br>baseline = 65%<br>boosted = 80%	 [15]<br>[14, 21]<br>[18]
		**omicron**<br>35%	 [22]
VE_I	Vaccine effectiveness to decrease infectiousness	**delta**<br>waning = 10%<br>baseline = 35%<br>boosted = 60%	 [21]<br>[14, 21]<br>[18]
		**omicron**<br>5%	 [22]
VE_P	Vaccine effectiveness to decrease disease progression to hospitalization given infection	**delta**<br>waning = 80%<br>baseline = 86%<br>boosted = 90%	 [22, 31, 61]<br>[22, 31, 61]<br>[22, 31]
		**omicron**<br>77%	 [22, 31]
XE_S	Infection-acquired immunity effectiveness to decrease susceptibility to infection	**delta**<br>63%	 [18]
		**omicron**<br>35%	 [23]
XE_I	Infection-acquired immunity effectiveness to decrease infectiousness	**delta**<br>13%	 [18]
		**omicron**<br>5%	 [23]
XE_P	Infection-acquired immunity effectiveness to decrease disease progression to hospitalizations given infection	**delta**<br>54%	 [23, 62]
		**omicron**<br>74%	 [23]
HE_S	Hybrid immunity effectiveness to decrease susceptibility to infection (vaccine- and infection-acquired immunity)	**delta**<br>waning = 81.5%<br>baseline = 87.1%<br>boosted = 92.6%	see Methods
		**omicron**<br>50%	—
HE_I	Hybrid immunity effectiveness to decrease infectiousness	**delta**<br>waning = 21.7%<br>baseline = 43.5%<br>boosted = 65.2%	see Methods
		**omicron**<br>10%	—
HE_P	Hybrid immunity effectiveness to decrease disease progression to hospitalizations given infection	**delta**<br>waning = 80%<br>baseline = 86%<br>boosted = 90%	see Methods
		**omicron**<br>77%	—

Supplementary Table 2: **Summary of immunity parameters used in modeling and simulation.**

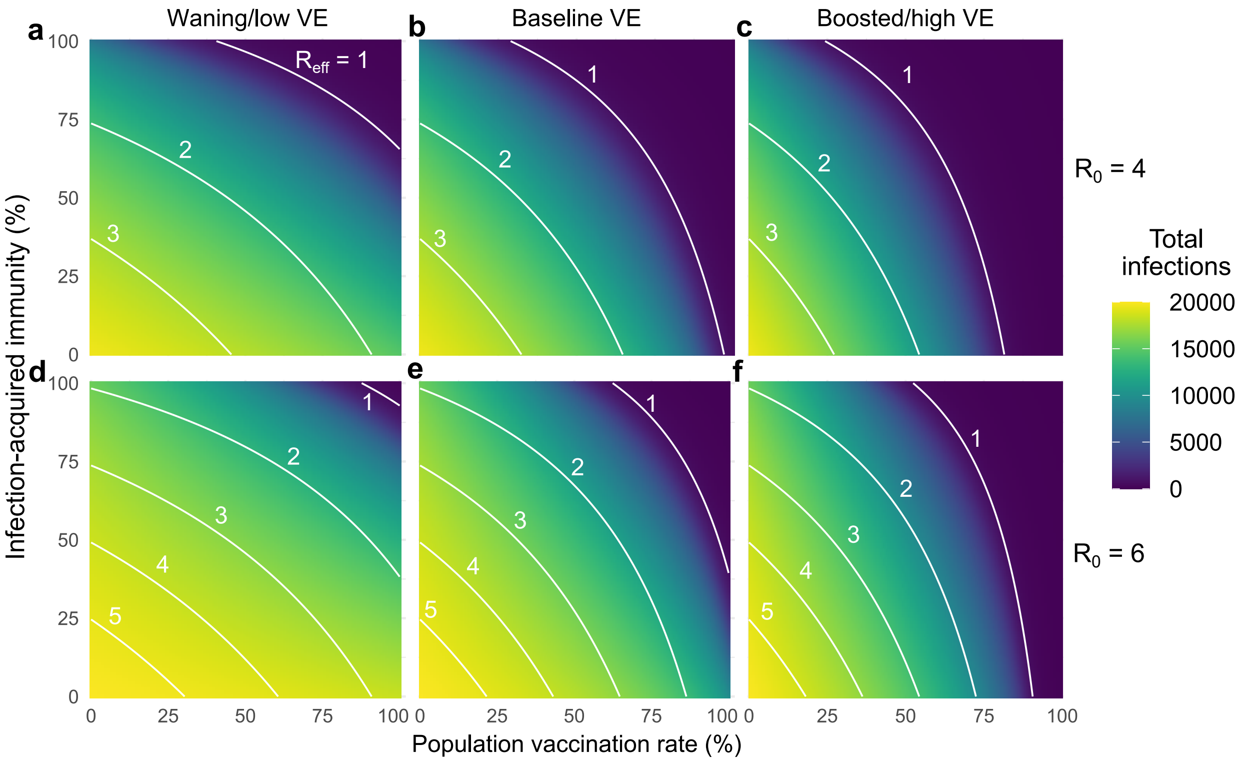

Supplementary Figure 1: **Vaccination's impact on the total number of infections depends on vaccine effectiveness and R_0^{NPI}.** For (top row) $R_0^{\text{NPI}} = 4$ and (bottom row) $R_0^{\text{NPI}} = 6$, heatmaps show the total number of infections as past infection and vaccination rates vary for vaccines with (a) waning, (b) baseline, and (c) boosted effectiveness vs the delta variant. See Supplementary Tables 1 and 2 for scenario parameter values. Curves denote the effective reproductive number R_{eff} at $t = 0$ as annotated. $N = 20,000$.

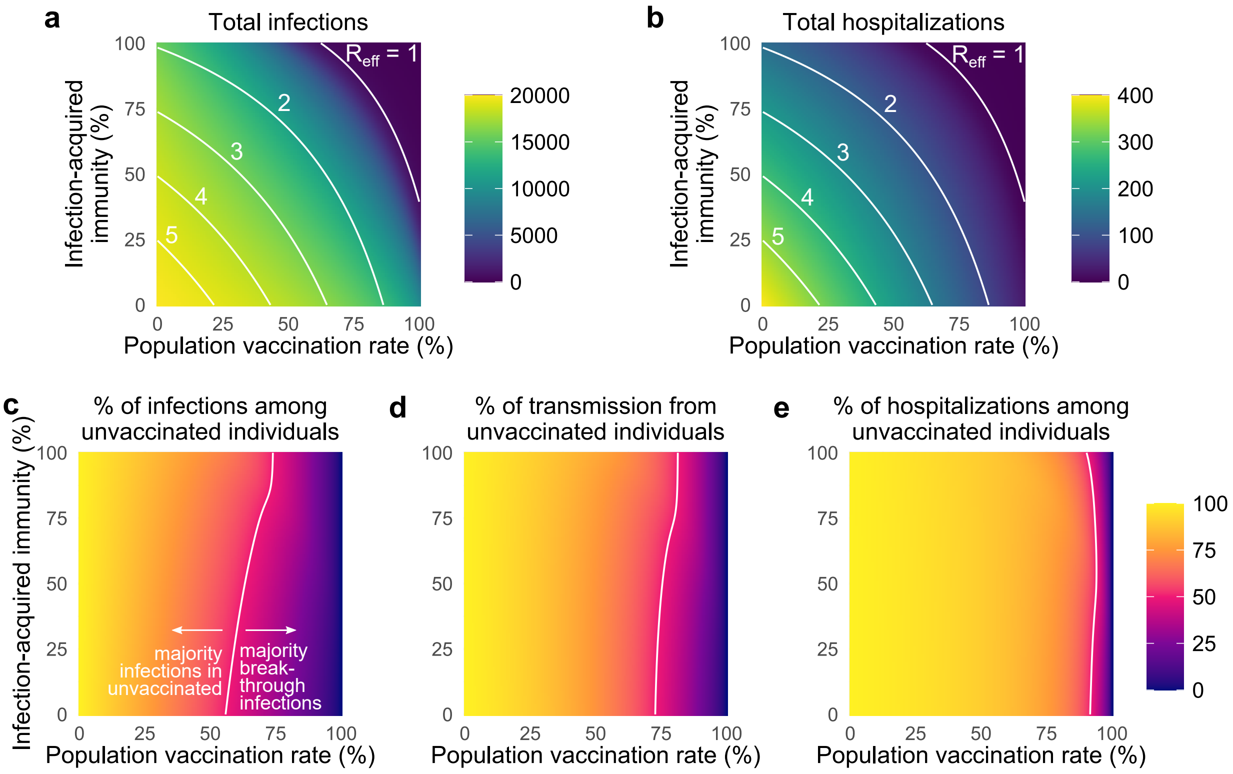

Supplementary Figure 2: **Vaccination and past infection affect epidemic potential, vaccine breakthroughs, and drivers of transmission**. (a) Curves denote the effective reproductive number R_{eff} at $t = 0$ as annotated, as past infection and vaccination rates vary. Heatmaps show (a) the total number of infections, (b) the percentage of total infections occurring in the unvaccinated population and (c) the percentage of total infections caused by the unvaccinated population. White annotation curves in (b) and (c) indicate the 50% point. $N = 20,000$ and $R_0^{\text{NPI}} = 6$ in all panels, with baseline VE and immunity parameters vs the delta variant; see Figure 2 for $R_0^{\text{NPI}} = 4$. See Supplementary Table 2 for values for infection-acquired, vaccine-acquired, and hybrid immunity parameters.

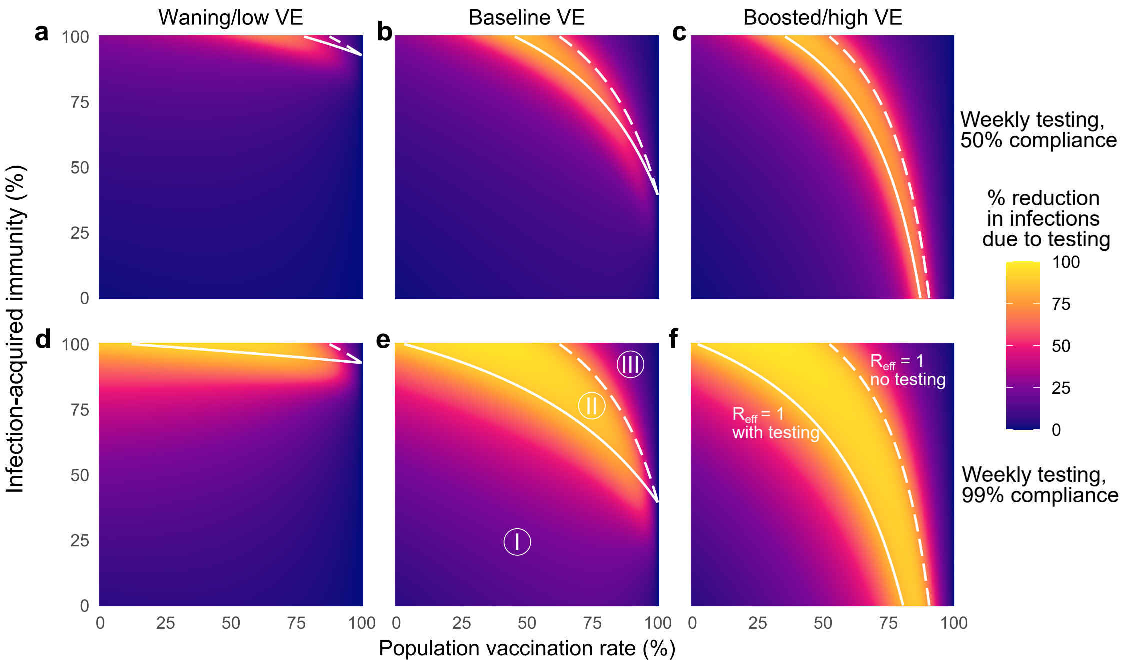

Supplementary Figure 3: **The impacts of unvaccinated-only screening on total infections depend on population immunity, compliance, and vaccine effectiveness.** Percent reduction in infections due to screening over various population vaccination rates assuming low (a, d), baseline (b, e), and high (c, f) vaccine effectiveness with once-weekly screening at 50% (top row) and 99% (bottom row) compliance. White lines indicate the population immunity rate at which $R_{\text{eff}} = 1$ with screening (solid) and without screening (dashed), which divide the space into three regions, labeled I, II and III. See Supplementary Table 2 for immunity parameter values. $R_0^{\text{NPI}} = 6$ in all panels; see Figure 5 for $R_0^{\text{NPI}} = 4$.

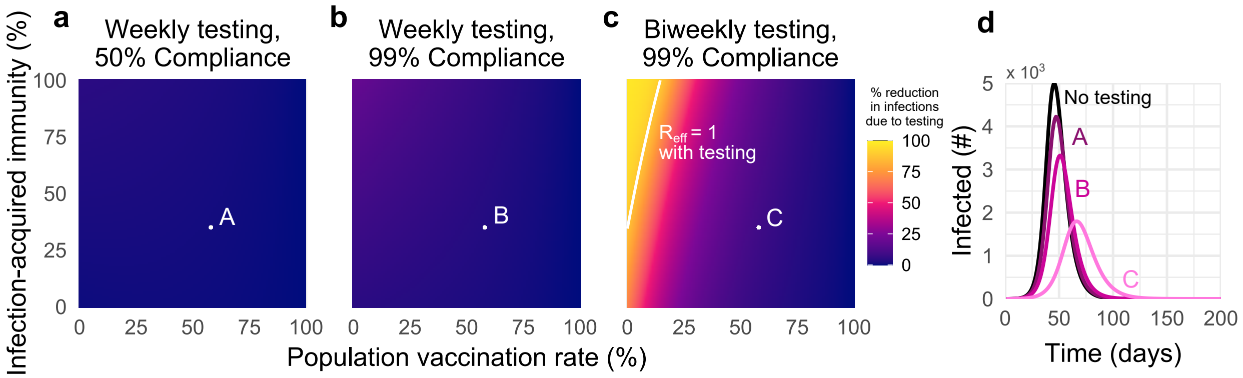

Supplementary Figure 4: **Unvaccinated-only screening during omicron transmission cannot achieve $R_{\text{eff}} < 1$ except in low-vaccination and high-frequency regimes.** Percent reduction in infections due to screening over various population vaccination rates assuming plausible parameters for immunity associated with prior infection, vaccination, or both, with (a) once-weekly screening at 50% compliance, (b) once-weekly screening at 99% compliance, and (c) twice-weekly screening at 99% compliance. (d) Number of individuals infected over time, under screening scenarios denoted A, B, C, compared with no screening (black) with 58% vaccination rate and 35% rate of prior infection. Solid white line indicates the population immunity combinations for which $R_{\text{eff}} = 1$ with screening; no combinations exist to produce $R_{\text{eff}} = 1$ without screening. See Supplementary Table 2 for immunity parameter values. $R_0^{\text{NPI}} = 6$ in all panels. See Figure 6 for $R_0^{\text{NPI}} = 4$.

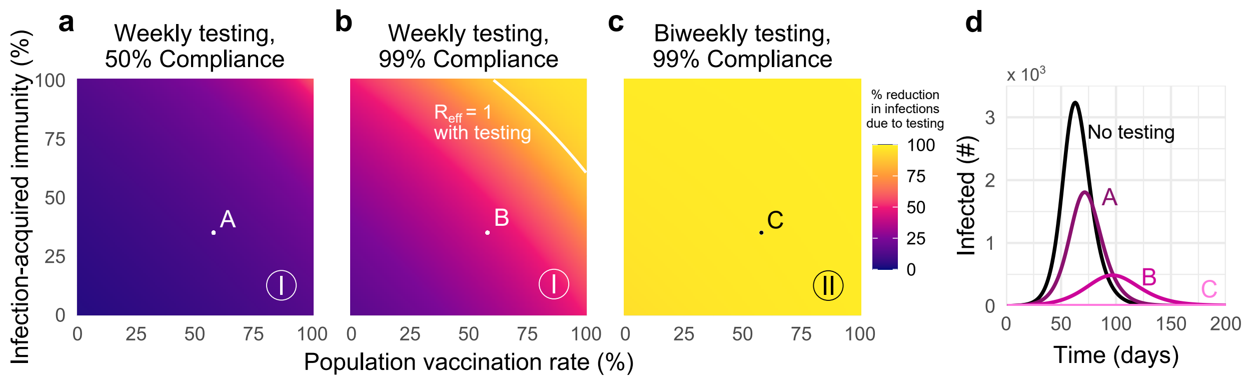

Supplementary Figure 5: **Universal testing during omicron transmission can achieve $R_{\text{eff}} < 1$ in high-compliance and high-frequency regimes.** Percent reduction in infections due to universal screening over various population vaccination rates assuming plausible parameters for immunity associated with prior infection, vaccination, or both, with (a) once-weekly screening at 50% compliance, (b) once-weekly screening at 99% compliance, and (c) twice-weekly screening at 99% compliance. (d) Number of individuals infected over time, under universal screening scenarios denoted A, B, C, compared with no screening (black) with 58% vaccination rate and 35% rate of prior infection. Solid white line indicates the population immunity combinations for which $R_{\text{eff}} = 1$ with screening; no combinations exist to produce $R_{\text{eff}} = 1$ without screening. See Supplementary Table 2 for immunity parameter values. $R_0^{\text{NPI}} = 4$ in all panels. See Figure 6 for unvaccinated-only screening.

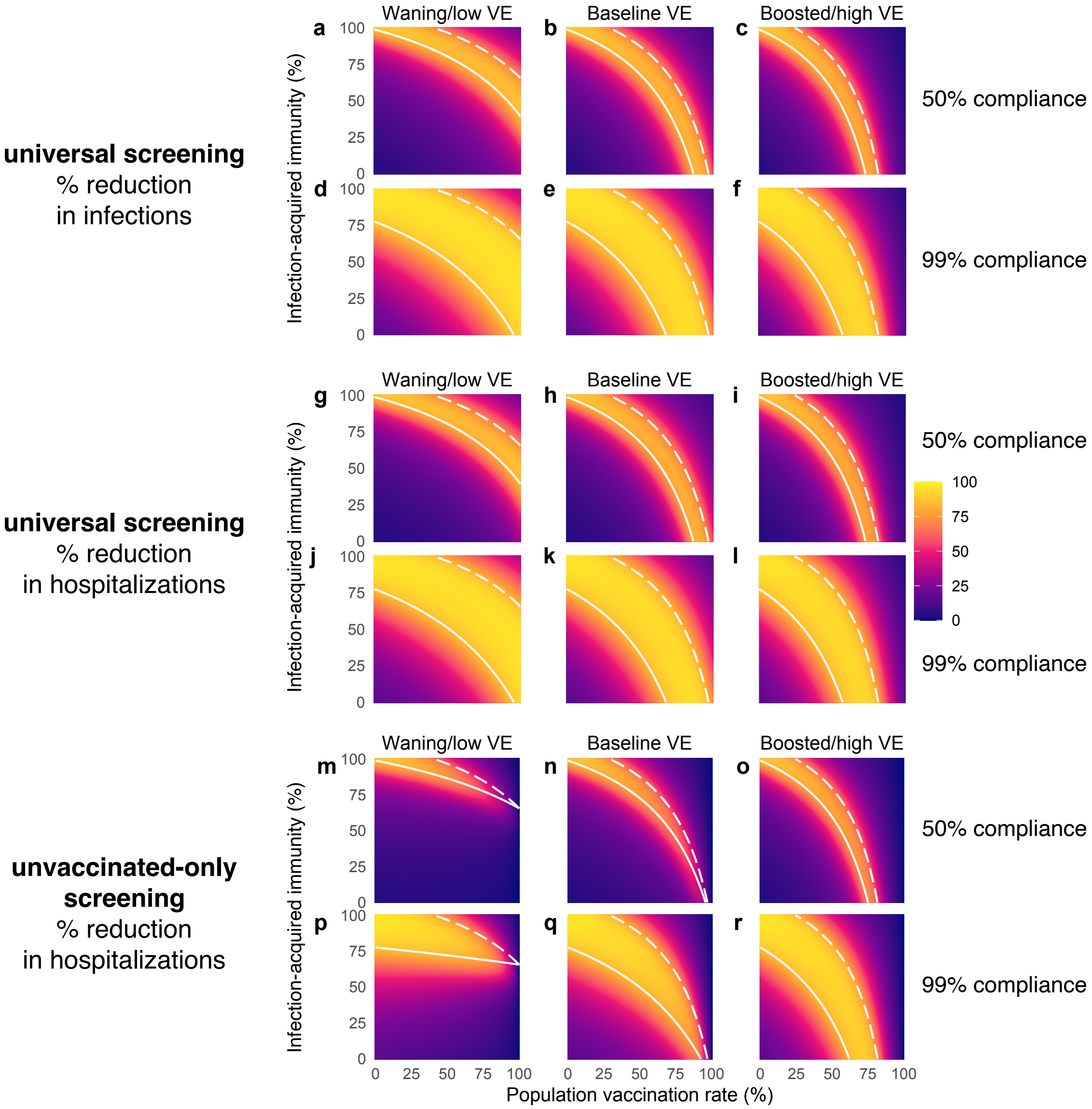

Supplementary Figure 6: **The impacts of screening on total infections and hospitalizations depend on population immunity, compliance, and vaccine effectiveness.** (Top/middle panel) Percent reduction in infections/hospitalizations due to universal screening over various population vaccination rates assuming low (a, d, g, j), baseline (b, e, h, k), and high (c, f, i, l) VE vs the delta variant with once-weekly screening at 50% (top row) and 99% (bottom row) compliance. (Bottom panel) Percent reduction in hospitalizations due to unvaccinated-only screening over various population vaccination rates assuming low (m, p), baseline (n, q), and high (o, r) VE vs the delta variant with once-weekly screening at 50% (top row) and 99% (bottom row) compliance. White lines indicate the population immunity rate at which $R_{\text{eff}} = 1$ with screening (solid) and without screening (dashed), which divide the space into three regions, labeled I, II and III. See Supplementary Table 2 for immunity parameter values. $R_0^{\text{NPI}} = 4$ in all panels; see Figure 5 for percent reduction in infections under unvaccinated-only testing.

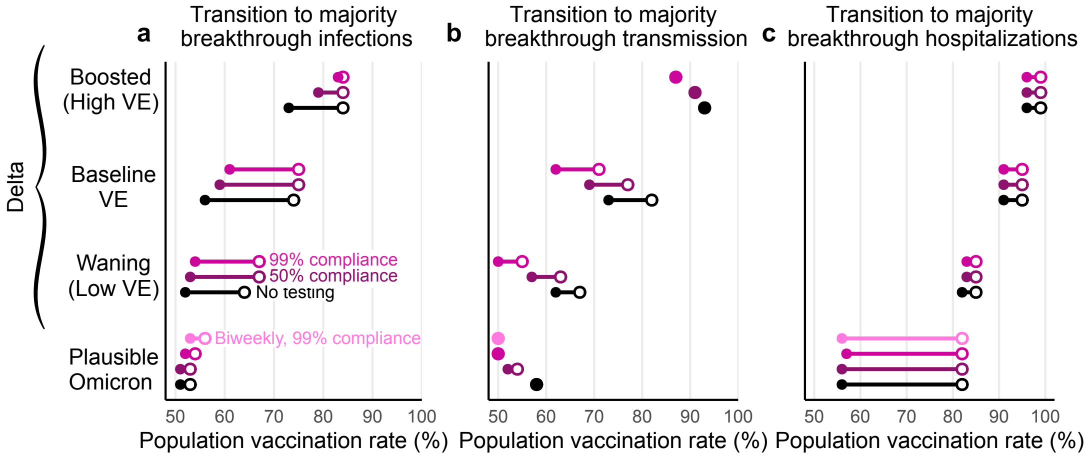

Supplementary Figure 7: **Screening via testing and vaccine effectiveness affect transition points to majority-breakthrough regimes.** The vaccination rates at which the vaccinated population makes up the majority of (a) infections and (b) transmission for low, moderate, and high vaccine effectiveness scenarios. Minimum (filled circle) and maximum (open circle) endpoints show the variation in transition points over all combinations of vaccination and prior infection rates for no screening (black), 50% compliance (purple), and 99% compliance (pink) over all possible values for past infection rates. $R_0^{\text{NPI}} = 6$ for all plots; see Figure 7 for $R_0^{\text{NPI}} = 4$.

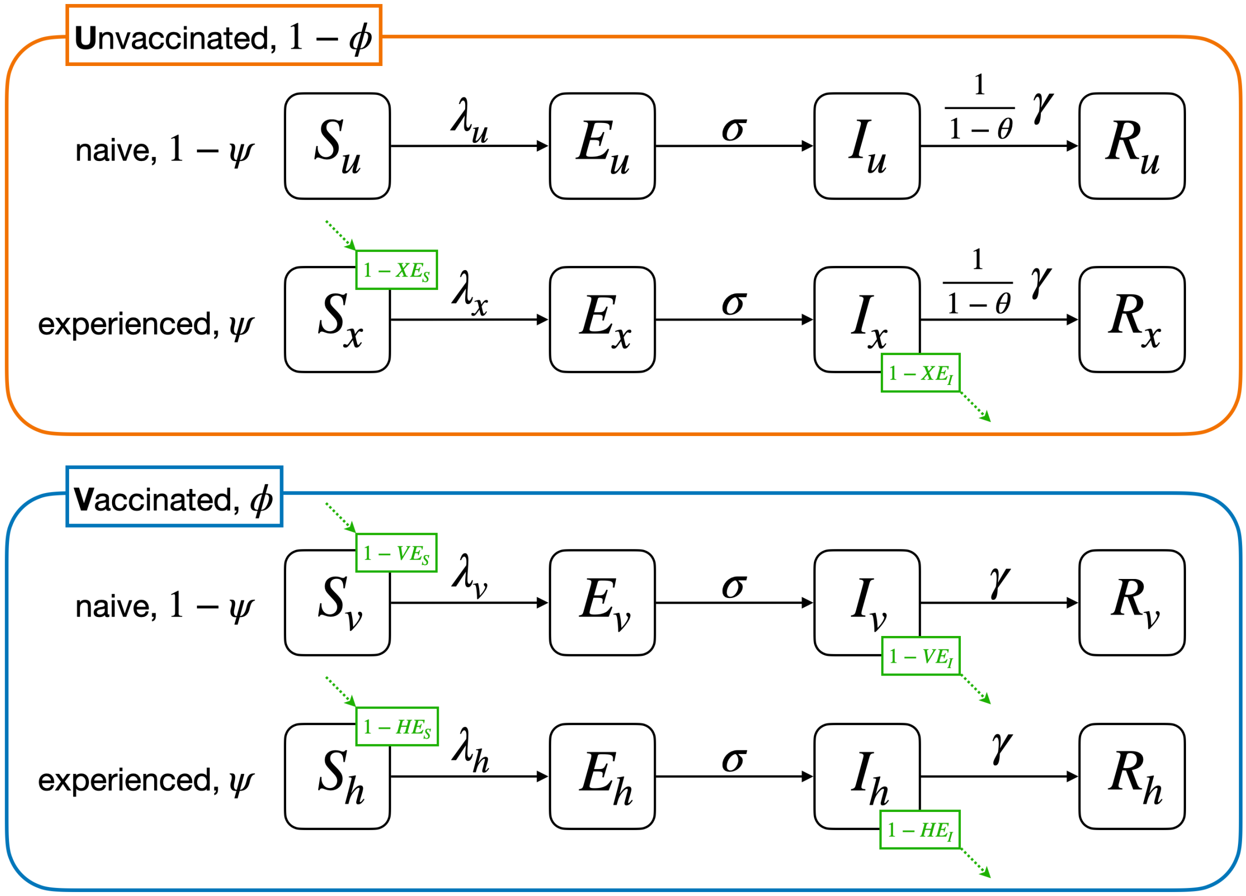

Supplementary Figure 8: **SEIR Model Flow Diagram.** SEIR model schematic depicting unvaccinated (u subscript), SARS-CoV-2 experienced (x subscript), vaccinated (v subscript), and both experienced and vaccinated ("hybrid"; h subscript) populations. Solid lines denote movement of individuals between classes at the given rate. The time spent infectious, $1/\gamma$, may be shortened by a factor of $1-\theta$ due to screening. Dashed lines denote infectious interactions, scaled by protection against infection ($\mathrm{VE}_S, \mathrm{HE}_S, \mathrm{XE}_S$) and transmission ($\mathrm{VE}_I, \mathrm{HE}_I, \mathrm{XE}_I$).

Derivation of effective reproductive number R_{eff}

With unvaccinated-only screening, this model's next generation matrix M, used to calculate the effective reproductive number R_{eff}, is given by

$$M = \frac{\alpha}{\gamma}\begin{pmatrix}1 & 0 & 0 & 0\\ 0 & 1-\text{XE}_S & 0 & 0\\ 0 & 0 & 1-\text{VE}_S & 0\\ 0 & 0 & 0 & 1-\text{HE}_S\end{pmatrix} C \begin{pmatrix}1-\theta & 0 & 0 & 0\\ 0 & (1-\text{XE}_I)(1-\theta) & 0 & 0\\ 0 & 0 & 1-\text{VE}_I & 0\\ 0 & 0 & 0 & 1-\text{HE}_I\end{pmatrix} \tag{S1}$$

where C is the contact matrix

$$C = \begin{pmatrix} c_{u\to u} & c_{x\to u} & c_{v\to u} & c_{h\to u}\\ c_{u\to x} & c_{x\to x} & c_{v\to x} & c_{h\to x}\\ c_{u\to v} & c_{x\to v} & c_{v\to v} & c_{h\to v}\\ c_{u\to h} & c_{x\to h} & c_{v\to h} & c_{h\to h}\end{pmatrix} \tag{S2}$$

with units of average number of contacts per person per day. In a well-mixed population of size N with proportions ϕ vaccinated and SARS-CoV-2 prior infection ψ,

$$C_{\text{well mixed}} = N\begin{pmatrix}\phi(1-\psi) & \phi\psi & (1-\phi)\psi & (1-\phi)(1-\psi)\\ \phi(1-\psi) & \phi\psi & (1-\phi)\psi & (1-\phi)(1-\psi)\\ \phi(1-\psi) & \phi\psi & (1-\phi)\psi & (1-\phi)(1-\psi)\\ \phi(1-\psi) & \phi\psi & (1-\phi)\psi & (1-\phi)(1-\psi)\end{pmatrix}. \tag{S3}$$

The effective reproductive number is the absolute value of the dominant eigenvalue of M. It depends on R_0^{NPI}, ϕ, and ψ, as well as values of VE, HE, XE, and θ,

$$R_{\text{eff}} = R_0^{\text{NPI}}\Big[\phi(1-\psi)(1-\text{VE}_S)(1-\text{VE}_I) + \phi\psi(1-\text{HE}_S)(1-\text{HE}_I) + (1-\phi)\psi(1-\text{XE}_S)(1-\text{XE}_I)(1-\theta) + (1-\phi)(1-\psi)(1-\theta)\Big]. \tag{S4}$$

This equation has the more compact form

$$R_{\text{eff}} = R_0^{\text{NPI}}\left[f_u(1-\theta) + f_x r_x(1-\theta) + f_v r_v + f_h r_h\right], \tag{S5}$$

after substituting $f_u = (1-\psi)(1-\phi)$, $f_x = \psi(1-\phi)$, $f_v = (1-\psi)\phi$, and $f_h = \phi\psi$, the fractions of the population in the unvaccinated, experienced, vaccinated, and hybrid immunity groups, respectively, and $r_x = (1-\text{XE}_I)(1-\text{XE}_S)$, $r_v = (1-\text{VE}_I)(1-\text{VE}_S)$, and $r_h = (1-\text{HE}_I)(1-\text{HE}_S)$, the cumulative impacts of transmission-related immunity on each group. Note that for universal screening testing, this equation becomes

$$R_{\text{eff}}^{\text{universal}} = R_0^{\text{NPI}}(1-\theta)\left[f_u + f_x r_x + f_v r_v + f_h r_h\right]. \tag{S6}$$

Setting $R_{\text{eff}} = 1$ in Eq. (S4) leads to the following required vaccination fraction ϕ to achieve a reproductive number below one,

$$\phi_{R=1} = \frac{(1-\theta)\left[\psi(1-\text{XE}_S)(1-\text{XE}_I) + (1-\psi)\right] - \frac{1}{R_0^{\text{NPI}}}}{(1-\theta)\left[\psi(1-\text{XE}_S)(1-\text{XE}_I) + (1-\psi)\right] - (1-\psi)(1-\text{VE}_S)(1-\text{VE}_I) - \psi(1-\text{HE}_S)(1-\text{HE}_I)}. \tag{S7}$$

In the absence of screening, this equation simplifies to

$$\phi_{R=1} = \frac{\psi(1-\mathrm{XE}_S)(1-\mathrm{XE}_I) + (1-\psi) - \frac{1}{R_0^{\mathrm{NPI}}}}{\psi(1-\mathrm{XE}_S)(1-\mathrm{XE}_I) + (1-\psi) - (1-\psi)(1-\mathrm{VE}_S)(1-\mathrm{VE}_I) - \psi(1-\mathrm{HE}_S)(1-\mathrm{HE}_I)}\,. \tag{S8}$$

[54] Melanie H. Chitwood, Ted Cohen, Kenneth Gunasekera, Joshua Havumaki, Fayette Klaassen, Nicolas A. Menzies, Virginia E. Pitzer, Marcus Russi, Joshua Salomon, Nicole Swartwood, Joshua L. Warren, and Daniel M. Weinberger. COVID estim - COVID-19 nowcasting. https://covidestim.org/, accessed Sept. 22, 2021.

[55] Nicholas Davies, Petra Klepac, Yang Liu, Kiesha Prem, Mark Jit, and Rosalind M Eggo. Age-dependent effects in the transmission and control of COVID-19 epidemics. *Nature Medicine*, 26(1205-1211), 2020.

[56] Bernard Cazelles, Benjamin Nguyen Van Yen, Clara Champagne, and Catherine Comiskey. Dynamics of the COVID-19 epidemic in Ireland under mitigation. *BMC Infectious Diseases*, 21(735), 2021.

[57] Scobie HM, Johnson AG, Suthar AB, et al. Monitoring incidence of COVID-19 cases, hospitalizations, and deaths, by vaccination status 13 U.S. jurisdictions, April 4July 17, 2021. *MMWR Morb Mortal Wkly Rep*, 70(1):2841290, 2021.

[58] Puranik A, Lenehan PJ, Silvert E, Niesen MJM, Corchado-Garcia J, O'Horo JC, Virk A, Swift MD, Halamka J amd Badley AD, Venkatakrishnan AJ, and Soundararajan V. Comparison of two highly-effective mRNA vaccines for COVID-19 during periods of alpha and delta variant prevalence. *medRxiv*, 2021.

[59] Ana Cecilia Ulloa, Sarah A. Buchan, Nick Daneman, and Kevin A Brown. Early estimates of sars-cov-2 omicron variant severity based on a matched cohort study, ontario, canada. *medRxiv*, 2022.

[60] Joseph A. Lewnard, Vennis X. Hong, Manish M. Patel, Rebecca Kahn, Marc Lipsitch, and Sara Y. Tartof. Clinical outcomes among patients infected with omicron (b.1.1.529) sars-cov-2 variant in southern california. *medRxiv*, 2022.

[61] Nick Andrews, Elise Tessier, Julia Stowe, Charlotte Gower, Freja Kirsebom, Ruth Simmons, Eileen Gallagher, Simon Thelwall, Natalie Groves, Gavin Dabrera, Richard Myers, Colin N.J. Campbell, Gayatri Amirthalingam, Matt Edmunds, Maria Zambon, Kevin Brown, Susan Hopkins, Meera Chand, Shamez N. Ladhani, Mary Ramsay, and Jamie Lopez Bernal. Duration of protection against mild and severe disease by Covid-19 vaccines. *NEJM*, 386(4):340–350, 2022.

[62] Bozio CH, Grannis SJ, Naleway AL, Ong TC, Butterfield KA, DeSilva MB, Natarajan K, Yang DH, Rao S, et al. Laboratory-confirmed COVID-19 among adults hospitalized with COVID-19-like illness with infection-induced or mRNA vaccine-induced SARS-CoV-2 immunity - nine states, January-September 2021. *MMWR Morb Mortal Wkly Rep.*, 70(44):1539–1544, 2021.
