# Supplementary material for: SARS-CoV-2 transmission and impacts of unvaccinated-only screening in populations of mixed vaccination status
Source: Nat Commun. 2022 May 19;13:2777. doi: 10.1038/s41467-022-30144-7 (PMC9120147; doi:10.1038/s41467-022-30144-7)
Supplement: Supplementary file 2 — Reporting Summary [file 41467_2022_30144_MOESM2_ESM.pdf]

nature portfolio

Corresponding author(s): Daniel Larremore

Last updated by author(s): Mar 23, 2022

Reporting Summary

Nature Portfolio wishes to improve the reproducibility of the work that we publish. This form provides structure for consistency and transparency in reporting. For further information on Nature Portfolio policies, see our Editorial Policies and the Editorial Policy Checklist.

Statistics

For all statistical analyses, confirm that the following items are present in the figure legend, table legend, main text, or Methods section.

n/a	Confirmed	
☐	☒	The exact sample size (*n*) for each experimental group/condition, given as a discrete number and unit of measurement
☒	☐	A statement on whether measurements were taken from distinct samples or whether the same sample was measured repeatedly
☒	☐	The statistical test(s) used AND whether they are one- or two-sided<br>*Only common tests should be described solely by name; describe more complex techniques in the Methods section.*
☒	☐	A description of all covariates tested
☒	☐	A description of any assumptions or corrections, such as tests of normality and adjustment for multiple comparisons
☒	☐	A full description of the statistical parameters including central tendency (e.g. means) or other basic estimates (e.g. regression coefficient) AND variation (e.g. standard deviation) or associated estimates of uncertainty (e.g. confidence intervals)
☒	☐	For null hypothesis testing, the test statistic (e.g. *F*, *t*, *r*) with confidence intervals, effect sizes, degrees of freedom and *P* value noted<br>*Give P values as exact values whenever suitable.*
☒	☐	For Bayesian analysis, information on the choice of priors and Markov chain Monte Carlo settings
☒	☐	For hierarchical and complex designs, identification of the appropriate level for tests and full reporting of outcomes
☒	☐	Estimates of effect sizes (e.g. Cohen's *d*, Pearson's *r*), indicating how they were calculated

Our web collection on statistics for biologists contains articles on many of the points above.

Software and code

Policy information about availability of computer code

Data collection	Data was generated from custom code defining an ordinary differential equations model of population transmission dynamics. Model equations were solved using lsoda solver from the package deSolve version 1.28, R version 4.1.0.
Data analysis	All code is open source and provided by the authors. Model simulation data used in this manuscript is also available. Both can be found in the in the open source Github repository at https://zenodo.org/badge/latestdoi/419096560

For manuscripts utilizing custom algorithms or software that are central to the research but not yet described in published literature, software must be made available to editors and reviewers. We strongly encourage code deposition in a community repository (e.g. GitHub). See the Nature Portfolio guidelines for submitting code & software for further information.

Data

Policy information about availability of data

All manuscripts must include a data availability statement. This statement should provide the following information, where applicable:

- Accession codes, unique identifiers, or web links for publicly available datasets
- A description of any restrictions on data availability
- For clinical datasets or third party data, please ensure that the statement adheres to our policy

Data sharing not applicable to this article as no datasets were generated or analysed during the current study.

Field-specific reporting

Please select the one below that is the best fit for your research. If you are not sure, read the appropriate sections before making your selection.

- [x] Life sciences
- [ ] Behavioural & social sciences
- [ ] Ecological, evolutionary & environmental sciences

For a reference copy of the document with all sections, see nature.com/documents/nr-reporting-summary-flat.pdf

Life sciences study design

All studies must disclose on these points even when the disclosure is negative.

Sample size	All example simulations employed a sample size of 20,000 individuals, with vaccination and prior infection rates reflecting best estimates as of November, 2021. The size of 20,000 was chosen to reflect a small town or large university campus; the mathematics and conclusions of this study are invariant to the choice of population size.
Data exclusions	No data were excluded from our simulation analyses.
Replication	The model implemented here is deterministic, so an identical implementation on a different machine would provide the same results. However, note that uncertainty exists in the parameter values informing the model, such as the effectiveness of vaccination against infection. Sensitivity analyses revealed these trends, but the system is sensitive to changing these parameter values and initial conditions.
Randomization	Because no treatment was being allocated across a heterogeneous population, randomization was not applicable in this simulation study.
Blinding	Because all experiments were simulation-based, with no treatment of subjects, blinding was not applicable.

Reporting for specific materials, systems and methods

We require information from authors about some types of materials, experimental systems and methods used in many studies. Here, indicate whether each material, system or method listed is relevant to your study. If you are not sure if a list item applies to your research, read the appropriate section before selecting a response.

Materials & experimental systems

n/a	Involved in the study	
☒	☐	Antibodies
☒	☐	Eukaryotic cell lines
☒	☐	Palaeontology and archaeology
☒	☐	Animals and other organisms
☒	☐	Human research participants
☒	☐	Clinical data
☒	☐	Dual use research of concern

Methods

n/a	Involved in the study	
☒	☐	ChIP-seq
☒	☐	Flow cytometry
☒	☐	MRI-based neuroimaging
